# Numerical Investigation on the Biomechanical Performance of Laparoscopic-Assisted Plate Used for Fixing Pelvic Anterior Ring Fracture

**DOI:** 10.1155/2017/9261037

**Published:** 2017-05-23

**Authors:** Yiqian He, Yongtao Lu, Baosheng Yin, Li Yu

**Affiliations:** ^1^State Key Lab of Structural Analysis for Industrial Equipment, Department of Engineering Mechanics, International Research Center for Computational Mechanics, Dalian University of Technology, Dalian 116024, China; ^2^Department of Orthopaedics, First Affiliated Hospital of Dalian Medical University, Dalian, Liaoning 116044, China

## Abstract

Because of the minimal soft tissue injury, the laparoscopic-assisted internal fixation is a promising technique in fixing the pelvic anterior ring fracture. The aim of this study was to investigate the biomechanical performance of the laparoscopic-assisted plate by the finite element method. Four kinds of implants were investigated, that is, the laparoscopic-assisted plate (LAP), the percutaneous anterior pelvic bridge (PAPB), the transramus intraosseous screw (TIS), and the open reduction (OR). The stability of the implants was investigated under three loading cases, showing that when the LAP was used, the stress at the fracture site was smaller than that at other parts, while for other implants, the high stress was always around the fracture site. In conclusion, the LAP demonstrated a good biomechanical performance in fixing the pelvic anterior ring fracture and is a promising technique in clinical applications.

## 1. Introduction

**Figure 1:**
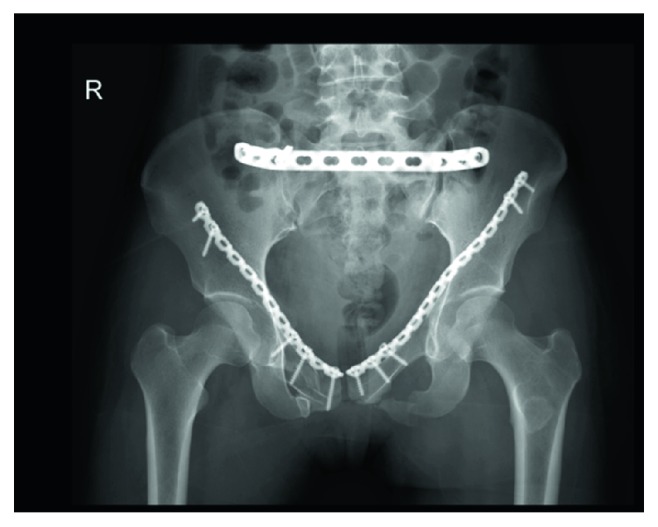
The X-ray image of the pelvis with the laparoscopic-assisted plate.

The pelvis is a bony structure complex, consisting of several segments that form a solid ring, which protects the organs (vascular structures, genital, urinary, and gastrointestinal) that it contains [1, 2]. The disruption of the pelvic ring often resulted from high-energy traumas, accounting for 3%-4% of all fractures, and has a high mortality of 19% [1, 2]. Pelvic anterior ring fixation methods, such as the formal open plating, external fixation, and transramus intraosseous screw fixation, are currently widely used in clinics [2]. Recently, a laparoscopic-assisted minimally invasive technique was developed for fixing pelvic ring fractures [3, 4]. This technique has many advantages, such as minimal soft tissue dissection, diminished blood loss, and reduced postoperative pain. Clinically, the authors' group has successfully completed one case using the laparoscopic-assisted plate (LAP) in treating a 35-year-old patient who had anterior pelvic ring fractures on both sides. The X-ray image after the operation is shown in Figure 1.

When the pelvic ring was fixed, a stable effect was expected by the surgeons. Therefore, the biomechanical stability is a key parameter for evaluating the performance of the implant. Generally, experimental testing and finite element analysis (FEA) are two widely used approaches to investigate the stability of the implants, but there are very limited investigations on the implants used in fixing pelvic anterior ring fractures. On the experimental side, Acklin et al.'s study is the only work which performed a mechanical testing on the plates and screws used in anterior pelvic ring fractures, and it was found that the plates were superior to the screws [5]. On the FE side, although FEA is proved to be an effective tool for choosing the best surgical method [6, 7], there is no previous numerical work on the investigation of the biomechanical stability of the implants used in fixing the pelvic anterior ring factures.

Giving the fact that the laparoscopic-assisted plate (LAP) is a new technique in fixing the pelvic anterior ring fracture and there is no investigation on the stability of the LAP, the aim of this study was to evaluate the biomechanical stability of the LAP and compare its stability with three other implant fixations, that is, the laparoscopic-assisted plate open reduction, percutaneous anterior pelvic bridge, and transramus intraosseous screw.

## 2. Material and Method

### 2.1. Clinical CT Scan

The CT scan of a 25-year-old healthy male was performed with a 64-Slice LightSpeed1 CT Scanner (Phillips, Netherlands). The CT data was obtained with an image interval of 1.0 mm and an image resolution of 0.69 × 0.69 mm^2^.

### 2.2. Finite Element Model of the Pelvis

**Figure 2:**
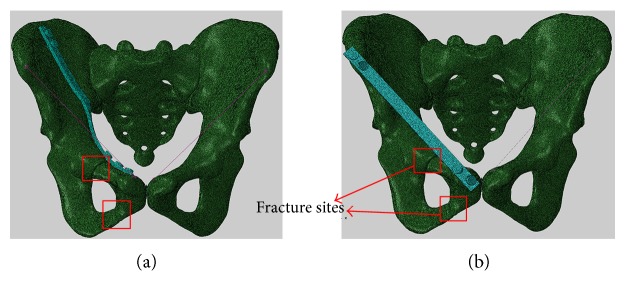
The finite element pelvic model with an anterior ring fracture fixed by a laparoscopic-assisted plate (LAP) (a) and a percutaneous anterior pelvic bridge (PAPB) (b).

The CT images were segmented through a semiautomatic process based on the pixel density using the image processing software Mimics (version 16, Software and Services for Biomedical Engineering, Materialise HQ, Belgium). The 3D geometry model was created and smoothed using the reverse engineering software Geomagic Studio (version 12, Raindrop Geomagic, NC, USA), and a bone fracture at the anterior ring of the pelvis was generated (Figure 2). The FE mesh was generated in the FE preprocessing software Hypermesh (version 13.0, Altair Engineering, Troy, MI, USA), and the FE mesh was imported to the FE analysis software Abaqus v.6.12 (version 6.12, Dassault Systemes Simulia Corp., Providence, RI, USA) for the calculations. The cortical bone of the pelvis was assumed to have a homogeneous thickness of 1.6 mm [8]. The FE model of the pelvis contains 349164 linear triangle elements (Type S3 in Abaqus). The ligaments in the pelvis were built by using spring elements in Abaqus (Figure 2). The connections between the synchondrosis pubis and bilateral pubis, as well as the sacrum-iliac (SI) joint facet, were fully constrained for simplification.

### 2.3. Finite Element Models of the Implants

#### 2.3.1. Laparoscopic-Assisted Plate (LAP)

As shown in Figure 2(a), a plate was fixed clinging to the medial ilium plate from iliac fossa to symphysis. Totally, five screws were fixed: two were placed at the locations with a distance of 10 mm to the fracture end, two were placed at the iliac crest with the distances of 25 mm and 15 mm to the fracture end, respectively, and the last screw was fixed at the pubic tubercle. The length, width, and height of the LAP were 167 mm × 10 mm × 3 mm, respectively.

#### 2.3.2. Percutaneous Anterior Pelvic Bridge (PAPB)

Four screws were used to fix the percutaneous steel plate between the anterior superior iliac spine and the pubic tubercle as shown in Figure 2(b). The length, width, and height of PAPB are 160 mm × 10 mm × 3 mm, respectively.

#### 2.3.3. Transramus Intraosseous Screw (TIS)

**Figure 3:**
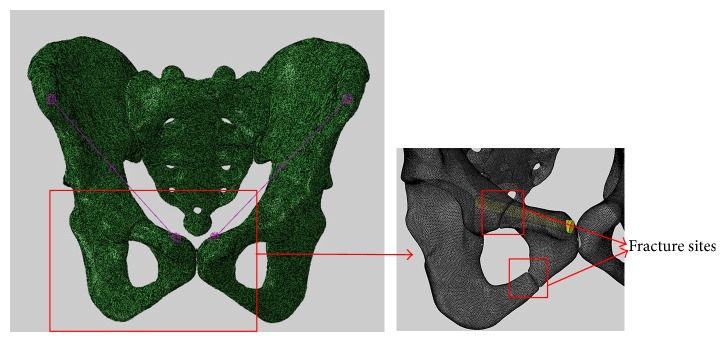
An FE model of the pelvis with a transramus intraosseous screw.

A transramus intraosseous screw was inserted into the pubic branch as shown in Figure 3. The length and diameter of the screw were 64 mm and 6.5 mm, respectively, and the diameter of the screw cap was 8 mm.

#### 2.3.4. Open Reduction (OR)

The fixation method was the same with the case for the laparoscopic-assisted minimally invasive plate as shown in Figure 2(a), but the inguinal ligament was cut off.

In building the finite element model for all the implants in this paper, quadratic tetrahedral elements are used with the element type C3D10 in Abaqus.

### 2.4. Boundary Conditions

**Figure 4:**
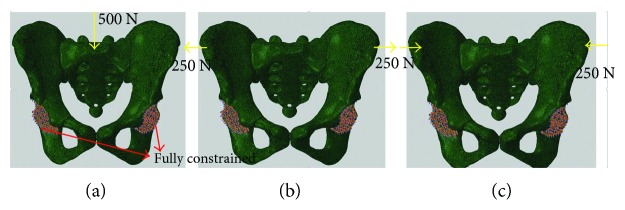
Boundary conditions applied on the pelvis, vertical force loading (a), open book-like force loading (b), and close book-like force loading (c).

In the generated FE models, the acetabulums were fully fixed and three loading cases were considered as described below (Figure 4):

1. Vertical force (VF): A vertical force of 500 N was applied on the surface of the sacrum (Figure 4) [8].
2. Open book-like force (OBLF): Two outward horizontal forces of 250 N were applied at both ends of the anterior superior iliac spine (Figure 4).
3. Close book-like force (CBLF): Two inward horizontal forces of 250 N were applied at both ends of the anterior superior iliac spine (Figure 4).

**Figure 5:**
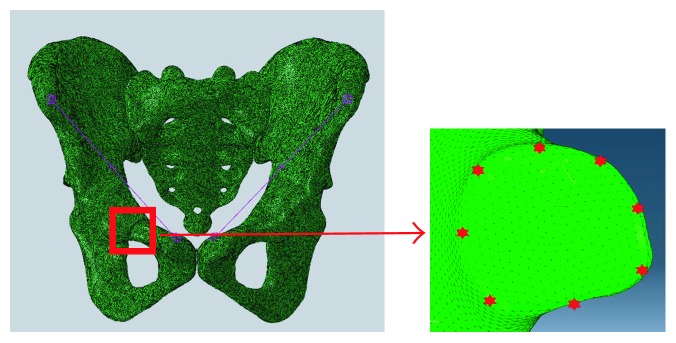
The points selected for investigation at the facture surface.

Because the interest of this study was on the deformation occurred at the fracture site, the values of displacements at the fracture site are calculated to characterize the stability of the pelvis with different implants. Eight points at the fracture end were selected, and the average resultant displacement of these points was calculated (Figure 5). First, the resultant displacement at each point was using the following formula:

(1) $$\begin{array}{c} u_{T}=\sqrt{u_{x}^{2}+u_{y}^{2}+u_{z}^{2}}, \end{array}$$

where *u*_*x*_, *u*_*y*_, and *u*_*z*_ are the displacements along the *x*‐, *y*‐, and *z*‐axes, respectively.

Then, the average resultant displacement was calculated as the average of the eight resultant displacements.

### 2.5. Material Parameters

Young's modulus and Poission's ratio of the cortical bones were set as *E*_*c*_ = 43530  MPa and *ν*_c_ = 0.2 [8]. The stiffness of the inguinal ligament was *k*_*l*_ = 250  N/mm [9]. Young's modulus and Poission's ratio of all implants are titanium alloy with *E*_*I*_ = 118.6 GPa and *ν*_c_ = 0.33 [10].

## 3. Results

### 3.1. The Average Displacement at the Fracture Site

Under the three loading cases, the average displacement at the fracture site is the smallest when the LAP was implanted followed by the TIS and PAPB. Under the vertical force, the average displacements at the fracture site are shown in Table 1. The average displacements of the LAP, PAPB, TIS, and OR are 0.0089 mm, 0.023 mm, 0.019 mm, and 0.0094 mm, respectively. Under the open book-like force load, the average displacements with fixation methods of the LAP, PAPB, TIS, and OR are 0.030 mm, 0.25 mm, 0.048 mm, and 0.029 mm, respectively (Table 1). Under the close book-like force loading, the average displacements are the same as the results with loading OBLF. The reason is that symmetric loading is applied, and only the scalar quantity of displacements is calculated without considering its direction.

### 3.2. The Distribution of von Mises Stresses at the Fracture Sites

**Figure 6:**
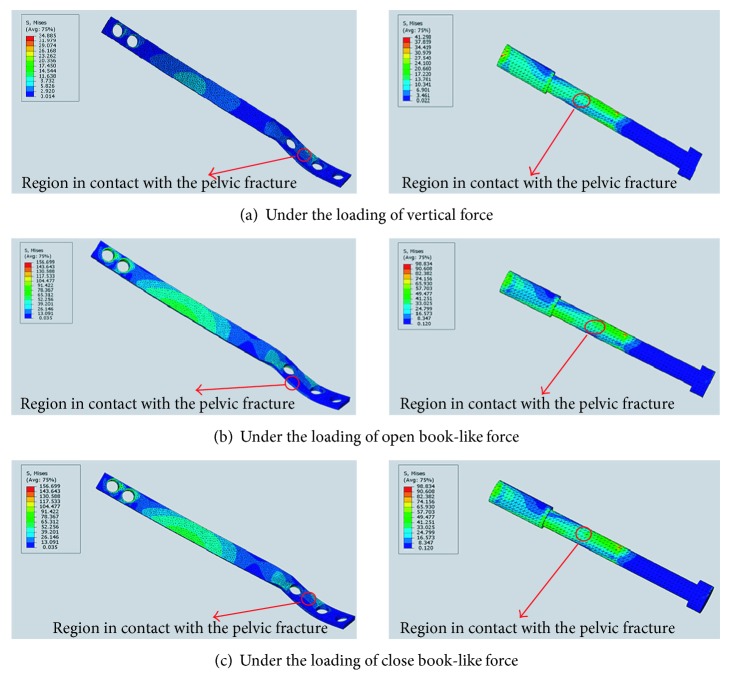
The distributions of von Mises stresses on the laparoscopic-assisted plate (left) and transramus intraosseous screw (right) under different loadings

In this part, only the LAP and the TIS are selected for comparing the stress distribution, as the PAPB produces worse stability as shown in the comparison of displacements at the fracture site, and the results of the displacement of the OR are similar with those of the LAP. The maximum stresses produced on the LAP are not always lower than those produced on the TIS. Under the vertical force, the maximum von Mises stresses are 34.885 MPa and 41.298 MPa for the LAP and TIS, respectively (Figure 6()). Under the open book-like force load, the maximum von Mises stresses are 156.699 MPa and 98.834 MPa for the LAP and TIS, respectively (Figure 6). Under the close book-like force loading, the von Mises stresses in the implants are the same as the case with loading OBLF.

The LAP improved the stress distribution. At the fracture site, the stress produced on the LAP is much lower than that produced on the TIS. On the other hand, for the LAP, the high stress occurred in the center location of the plate and not at the fracture site, while for the TIS, the high stress always occurred at the fracture site. Therefore, the LAP is superior to the TIS for fixing the pelvic anterior ring fracture.

## 4. Discussion

The pelvic anterior ring fracture is a serious public problem that is always involving pubic symphysis, usually causing the failure of the pelvis and the fracture at the pelvic posterior ring. Surgical treatment with internal fixations is widely used in the treatment of the pelvic anterior ring fracture. The traditional open reduction method usually uses ilioinguinal operative approach, which can sufficiently expose surgical field including the quadrilateral surface of the upper and lower surface of the pubic branch. Because the anatomic relationship is complicated in this region, the intraoperative injury involving important vessels and nerves could occur, including iliac external vascular injury, iliac external vascular thrombosis, femoral nerve injury, femoral lateral skin nerve injury, inguinal hernia, lymph leakage, and infection [11].

Hirvensalo et al. firstly introduced the “Stoppa” approach for complex hernia repair in the treatment of a pelvic fracture in 1993 [12]. By cutting rectus abdominis muscle at the vertical center of the inferior belly, the pelvic structure was sufficiently exposed so that the operative field was much clearer [13, 14]. Although the “Stoppa” approach is superior to the ilioinguinal approach, some complications could still happen, including peritoneal perforation, vascular nerve injury, deep vein thrombosis, pulmonary embolism, inguinal hernia, incision infection, and traumatic arthritis [15]. Hence, the application of this approach is very limited because of possible trauma and complications.

Cole et al. used the percutaneous anterior pelvic bridge to deal with the fracture of the pelvic anterior ring in 2012 [15, 16]. The operation made incision over the iliac crest and the pubic bone, inserting a plate from a subcutaneous channel upon the inguinal ligament, and then screws were fixed on the iliac crest and the tuberculum pubicum. The characteristics of this technique were that the plate was located upon the subcutaneous inguinal ligament like a “bridge” over the anatomical structures including lateral femoral cutaneous nerve, ilioinguinal nerve, iliohypogastric nerve, and femoral artery, femoral vein, and femoral nerve. In this method, the fracture reduction was not necessary, so only a simple operation with a short time was required, and bleeding was less [16]. From the biomechanical stability point of view, the FEA results presented in this paper showed that this operation method performed worse than the implants with the TIS and LAP under the three kinds of external loadings. In addition, there are inevitable complications because of the involvement of skin impact, pain, and subcutaneous placement.

With the development of imaging technology, some minimally invasive internal fixation techniques have been proposed. Among these methods, the transramus intraosseous screw method has advantages of small surgical trauma, less bleeding, and good posture adaptability [11]. The results of FE analysis show that there is relatively significant stress concentration under vertical stress and open book stress, which could increase the risk of breaking the screw. Hence, this technique may be clinically suitable for the close book anterior ring pelvic fracture, while not suitable for pubic branch stenosis or deformity cases. Furthermore, this method is demanding for navigation system, and there are still some other risks including a screw into the hip joint, screw deformation and breakage, nerve and blood vessel injury.

Zobrist et al. firstly reported two laparoscopic-assisted plate (LAP) cases in fixing the anterior pelvic ring fracture in 2002 [3]. Compared with other internal fixation methods, the LAP method has many advantages in fixing the pelvic anterior ring fracture. First, it builds pneumoperitoneum in the peritoneal clearance instead of cutting off the rectus abdominis, and thus the entire superior ramus of the pubis, pubic symphysis, and external iliac vessels can be clearly exposed. Second, under the view of endoscopy, part of the iliopectineal fascia is cut off to connect the iliac wing plate and the inner pelvic clearance, and a plate is inserted clinging to the surface of the bone, avoiding cutting off the rectus abdominis. Therefore, the LAP method can effectively prevent the cutaneous nerve injury and incisional hernia. The fracture reduction and internal fixation can be completed under a direct vision.

The FE results in this paper showed that the LAP has superior stability compared to the PAPB and TIS methods. Therefore, the LAP method is very promising in the treatment of the pelvic anterior ring fracture. However, it should be noted that the clinical experience of laparoscopic learning curve is long, and a good anatomical basis and open surgery experience are needed; the operation time in the initial stage is also long. Compared with the open reduction, the complications of the minimally invasive plate are significantly less, and the stability of the fixation is similar.

Due to the complexity of the human pelvis, some simplifications were made in the FE models and thus some limitations should be noted in this study. First, the cortical thickness was assumed to be constant with a value of 1.6 mm in the FE models. Considering that the aim of this study was to compare the biomechanical stability of different implants in the same pelvis, the implication made in the FE model was reasonable. Second, bilateral inguinal ligaments were simulated according to the simplification in this paper, and the connections between the synchondrosis pubis and bilateral pubis, as well as the sacrum-iliac (SI) joint facet were fully constrained in Abaqus. Considering that the forces were mainly transferred through the cortex and implant and the deformation was small (in linear elastic region), the simplification on the ligament modeling is reasonable. Last but not least, the positions of the fixed screws on the LAP or PAPB plate could make some influences on the results. The influence of screw positions will be investigated in the further work.

## 5. Conclusion

The biomechanical stabilities of four kinds of internal fixation methods for the anterior pelvic ring fracture were numerically investigated by the finite element method. The results showed that the laparoscopic-assisted plate (LAP) performed the best in the treatment of the pelvic anterior ring fracture, followed by the transramus intraosseous screw (TIS), while the percutaneous anterior pelvic bridge (PAPB) was the worst. Obviously, considering the good performance of the LAP method in biomechanical stability and other advantages of this method in clinical operations, it can be expected that the LAP method will have a good application prospect for treating the pelvic anterior ring fracture.

## Figures and Tables

**Table 1:** Node resultant displacements and the average value (mm) at the fracture site under different loading cases.

	N 1	N 2	N 3	N 4	N 5	N 6	N 7	N 8	Average
Under the loading of vertical force									
LAP	0.01	0.011	0.0092	0.0072	0.0086	0.007	0.0064	0.012	0.0089
PAPB	0.027	0.022	0.019	0.02	0.027	0.025	0.023	0.026	0.0230
TIS	0.025	0.0099	0.023	0.012	0.017	0.026	0.016	0.027	0.019
OR	0.011	0.012	0.0095	0.0074	0.009	0.0072	0.0066	0.013	0.0094
Under the loading of open book-like force									
LAP	0.031	0.036	0.033	0.029	0.026	0.025	0.026	0.036	0.030
PAPB	0.23	0.25	0.22	0.22	0.24	0.27	0.27	0.26	0.250
TIS	0.059	0.028	0.052	0.033	0.041	0.062	0.042	0.065	0.048
OR	0.031	0.037	0.033	0.027	0.024	0.023	0.023	0.037	0.029
Under the loading of close book-like force									
LAP	0.031	0.036	0.033	0.029	0.026	0.025	0.026	0.036	0.030
PAPB	0.23	0.25	0.22	0.22	0.24	0.27	0.27	0.26	0.250
TIS	0.058	0.028	0.052	0.033	0.041	0.062	0.042	0.065	0.048
OR	0.029	0.037	0.032	0.026	0.022	0.021	0.022	0.0365	0.028
